# Gene expression profile analysis identifies metastasis and chemoresistance-associated genes in epithelial ovarian carcinoma cells

**DOI:** 10.1007/s12032-014-0426-5

**Published:** 2014-12-11

**Authors:** Liancheng Zhu, Zhenhua Hu, Juanjuan Liu, Jian Gao, Bei Lin

**Affiliations:** Department of Obstetrics and Gynecology, Shengjing Hospital Affiliated to China Medical University, Shenyang, 110004 Liaoning Province China

**Keywords:** Gene expression profile, Metastasis-associated gene, Chemoresistance-associated genes, Microarray, Epithelial ovarian carcinoma, FOXP1

## Abstract

The purpose of this study was to identify genes that associated with higher ability of metastasis and chemotherapic resistance in epithelial ovarian carcinoma (EOC) cells. An oligonucleotide microarray with probe sets complementary to 41,000^+^ unique human genes and transcripts was used to determine whether gene expression profile may differentiate three epithelial ovarian cell lines (RMG-I-C, COC1 and HO8910) from their sub-lines (RMG-I-H, COCI/DDP and HO8910/PM) with higher ability of metastasis and chemotherapic resistance. Quantitative real-time PCR and immunohistochemical staining validated the microarray results. Hierarchic cluster analysis of gene expression identified 49 genes that exhibited ≥2.0-fold change and *P* value ≤0.05. Highly differential expression of GCET2, NLRP4, FOXP1 and SNX29 genes was validated by quantitative PCR in all cell line samples. Finally, FOXP1 was validated at the protein level by immunohistochemistry in paraffin embedded ovarian tissues (i.e., for metastasis, 15 primary EOC and 10 omental metastasis [OM]; for chemoresistance, 13 sensitive and 13 resistant EOC). The identification of higher ability of metastasis and chemotherapic resistance-associated genes may provide a foundation for the development of new type-specific diagnostic strategies and treatment for metastasis and chemotherapic resistance in epithelial ovarian cancer.

## Introduction

Epithelial ovarian cancers (EOC) are high aggressive tumors associated with high mortality and morbidity in gynecology. Although the 5-year survival rate is 90 % for women with early-stage ovarian cancer and postoperative introduction of paclitaxel drug have improved the 5-year survival rate for advanced-stage ovarian cancer, patients with this cancer have a 5-year survival rate of only 30 % [1]. Standard therapy includes cytoreductive surgery with first-line combination chemotherapy, 75 % of patients initially respond to conventional chemotherapy; however, 80 % of these women eventually relapse and die from chemotherapy resistant disease. Thus, to understand the molecular basis of epithelial ovarian cancer metastasis and chemotherapeutic resistance is of vital importance and may have the potential to improve significantly the development of more specific and effective treatment against EOC. Comprehensive, high-throughput technologies such as gene expression microarrays have provided powerful tools for this purpose.

In the present study, the whole human genome oligo microarray was used to investigate the differential expression genes (DEGs) in the human ovary cancer cell lines RMG-1-C, COC1, HO8910 and their high malignant and chemoresistant sub-cell lines RMG-I-H, COC1/DDP and HO8910/PM. Hierarchical clustering of genes by the expression level of the DEGs was performed. The potential functions of the DEGs were analyzed by Gene Ontology (GO) and pathway enrichment analyses. In addition, the interaction relationships between these DEGs were investigated by regulatory network. We hope these metastasis and chemotherapy resistance-associated genes may be used for early detection of epithelial ovarian cancer and for development of more specific chemotherapy drugs against EOC.

## Materials and methods

### Cell culture

The human ovarian cancer cell strain RMG-1 was the courtesy of Doctor Iwamori Masao of Kinki University in Japan. We transfected the gene of extrinsic α1,2-fucosyl transferase (α1,2-FT) into RMG-1 to create cell line RMG-1-H with high expression of Lewis (y) and α1,2-fucosyl transferase [2, 3], and we discovered that compared with the empty plasmid vector transfected cell line RMG-1-C, cell line RMG-1-H showed enhanced cellular malignant biological behaviors, such as enhanced metastasis and proliferation [4], adhesion [5] and multiple drug resistance [6].

Human ovarian cancer cell lines HO-8910 and HO-8910 PM (a highly metastatic cell line derived from HO-8910) were purchased from the Cell Bank of Type Culture Collection of the Chinese Academy of Sciences (Shanghai, China) [7], human ovarian cancer cell lines COC1 and COC1/DDP (a platinum resistance cell line derived from COC1) were purchased from the China Center for Type Culture Collection (Wuhan, China). Cells were cultured in RPMI-1640 medium supplemented with 100 units/mL penicillin/streptomycin and 10 % fetal bovine serum (FBS) and maintained in an incubator at 37 °C under a humidified atmosphere of 5 % CO_2_. COC1/DDP cells were cultured in RPMI-1640 medium containing 0.5 µg/mL cisplatin (Sigma, St. Louis, MO, USA) to maintain the drug resistant phenotype. The cell lines and labels in this experiment are listed in Table 1.

**Table 1:** Cell line samples description

Label	A	B	C	1	2	3
Cell line	RMG-I-H	COC1/DDP	HO8910/PM	RMG-I-C	COC1	HO8910

### Total RNA extraction and gene chip hybridization

Total RNA was extracted from all 6 cell line samples with TRIZOL reagent (Life Technologies, Inc, Carlsbad, CA) and further purified with RNeasy Min-elute Clean-up Columns (Qiagen, Valencia, CA), as described by the manufacturers. Optical density for each sample of RNA was measured at OD 260 nm and OD 280 nm using NanoDrop ND-1000 (NanoDrop Technologies, Wilmington, DE, USA). All RNA samples isolated OD260/280 ratio should be close to 2.0 for pure RNA (ratios between 1.8 and 2.1 are acceptable). The OD A260/A230 ratio should be more than 1.8. Each isolated RNA sample was subjected to further quality check to ensure integrity of RNA with Agilent RNA 6000 Nano LabChip using Agilent 2100 Bioanalyzer (Agilent Technologies, Santa Clara, CA, USA). All RNA samples were verified to be intact with distinct 28S and 18S RNA bands at a ratio of approximately 2:1 and a RNA integrity number (RIN) > 7.

The samples were amplified and labeled using the Agilent Quick Amp labeling kit and hybridized with Agilent whole genome oligo microarray in Agilent’s SureHyb Hybridization Chambers in accordance with the manufacturer’s instructions. This array contains 41,000^+^ unique human genes and transcripts represented, all with public domain annotations, content sourced from RefSeq, Goldenpath Ensembl Unigene Human Genome (Build 33) and GenBank databases, over 70 % of the represented probes are validated by Agilent’s laboratory validation process, 4 × 44 K slide formats printed using Agilent’s 60-mer SurePrint technology. After hybridization and washing, the processed slides were scanned with the Agilent DNA microarray scanner (part number G2505B) using settings recommended by Agilent Technologies.

### Data analysis and clustering

Agilent Feature Extraction Software (version 10.5.1.1) was used to extract the signal intensity values from each gene chip, and the resulting text files were imported into the Agilent GeneSpring GX software (version11.0) for further analysis. The 6-microarray data sets were normalized in GeneSpring GX using the Agilent FE one-color scenario (mainly median normalization), and genes marked present or marginal in all samples were chosen for data analysis. DEGs were identified through fold-change screening comparing between cell lines group A, B, C and cell lines group 1, 2 and 3. The threshold used to screen up or down-regulated genes is fold change ≥2.0 and *P* value ≤0.05. A scatter plot was made to visualizingly assess the variation between chips. A hierarchical clustering and volcano plot were performed to visualizingly show a distinguishable gene expression profiling among samples.

### Validation for gene expression by quantitative real-time polymerase chain reaction

Real-time polymerase chain reaction (RT-PCR) was performed in triplicate with primer sets and probes that were specific for 4 selected genes that were found to be significantly differentially expressed. These 4 genes were 2 up-regulated genes: *GCET2, CFTR* and 2 down-regulated genes: *FOXP1, GARS*. cDNA was synthesized using random primers (hexamers) and Oligo 18dT and Superscript II Reverse Transcriptase (Invitrogen). Real-time PCR was performed on Roche LightCycler 480 sequence detection system, using the following amplification conditions: 5 min, 95 °C; followed by 40 cycles of 15 s 95 °C, 1 min 60 °C and 20 s 72 °C. CT values were determined using the IQ5 software (Bio-Rad). The primers mostly were searched from PrimerBank (http://pga.mgh.harvard.edu/primerbank/ index.html). Primers of target genes are listed in Table 2. The comparative threshold cycle method was used for the calculation of amplification fold, as specified by the manufacturer. The housekeeping gene glyceraldehyde-3-phosphate dehydrogenase (GAPDH) was used to normalize the quantity of complementary DNA that was used in the PCR reactions.

**Table 2:** Gene-specific primers used for validation

Gene name	GenBank accession number	Primer sequence (5′-3′)	Amplicon size (bp)
*GCET2*	NM_152785	F: *ACCCTCATCAATCATCGGGTT*	122
		R: *TCAGTCTCAGTTCCTCCCAAG*	
*CFTR*	NM_000492	F: *TGCCCTTCGGCGATGTTTTT*	127
		R: *GTTATCCGGGTCATAGGAAGCTA*	
*FOXP1*	NM_032682	F: *TCCCGTGTCAGTGGCTATGAT*	226
		R: *CTCTTTAGGCTGTTTTCCAGCAT*	
*GARS*	NM_002047	F: *TTGGCCCAGCTTGATAACTATG*	103
		R: *ACACTGGAGGGGATAGATCATTT*	
*GAPDH*	NM_001256799	F: *ACAACTTTGGTATCGTGGAAGG*	101
		R: *GCCATCACGCCACAGTTTC*	

*F* forward primer, *R* reverse primer

### Immunohistochemistry on paraffin embedded tissues

To evaluate protein expression levels for 1 of the 49 genes that was found to be different regulated, in consideration of a further study, immunohistochemical staining for FOXP1 was performed on ovarian tissue samples. 29 cases of primary ovarian cancer samples and 25 cases of omental metastatic (OM) ovarian cancer samples were collected from Shengjing Hospital of China Medical University in 2013, the histopathological diagnoses were determined using the WHO criteria. In our previous studies, we have established a set of ovarian cancer chemotherapeutic sensitive and resistant paraffin embedded samples [8, 9], and we randomly selected 40 samples in sensitive and 30 samples in resistant group for FOXP1 staining. There was no statistical difference between these two groups of ovarian samples in age, pathological subtype, lymph node metastasis or residual tumor size (data not shown). FOXP1 staining was performed using JC12 mouse anti-human monoclonal antibodies (diluted 1:40, JC12 was kindly provided by Alison H. Banham, University of Oxford, UK [10]) using the Envision detection kit (Maixin. Bio China). Positive myoepithelial cell staining and negative stromal cell staining in breast carcinoma were used as internal positive and negative controls, respectively. FOXP1 nuclear expression was scored using the following system: negative = 0; weak/focal = 1; strong focal/widespread moderate staining = 2; or strong/widespread staining = 3. Tumors that scored 2 or 3 were considered positive for FOXP1 nuclear staining. Survival analysis was performed on those patients, and the overall survival (OS) time was defined from the date of surgery (earliest was in July, 2004) to the date of death or the last follow-up (Jun, 2014).

### Enrichment analysis of DEGs

Gene Set Analysis Toolkit (Gestalt) tool was used to do enrichment analysis on the DEGs in three sections of function, biological process and molecular composition. Gestalt is a suite rich of analysis of biologically relevant content collecting eight species, including human, rat, mouse and other data from various different public data resources, such as NCBI, Ensemble, Gene Ontology (GO) and Kyoto Encyclopedia of Genes and Genomes (KEGG).

### Construction of gene regulatory network

The gene regulatory network was visualized by Cytoscape [11]. Proteins in the network served as the “nodes,” and each pairwise protein interaction (referred to as edge) was represented by an undirected link. The property of the network was analyzed with the plug-in network analysis.

### Statistics

Statistical analysis was performed using Graphpad Prism 6.0e Software for Mac OS X (GraphPad Software, La Jolla California USA, www.graphpad.com). Student’s *t* test was employed for comparison between two groups and one-way ANOVA with Tukey’s post hoc test was used for comparison between more than two groups. As to the analysis of quantitative RT-PCR result, data were expressed as mean ± SEM to compare on mRNA expression between different groups. The Chi square and Kaplan–Meier survival analysis were applied to analyze the nuclear expression of FOXP1. For these tests, a *P* value of <0.05 was considered statistically significant.

## Results

### Gene expression analysis and clustering

**Fig. 1:**
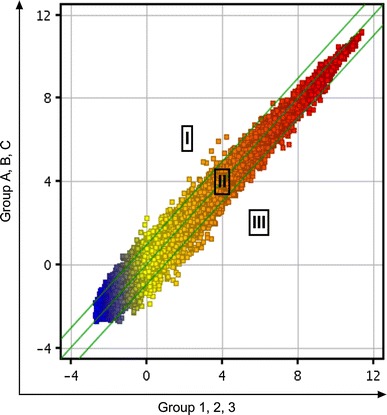
Representative *scatter plot* of changes in gene expression levels. *Scatter plot* is a visualization that is useful for assessing the variation (or reproducibility) between chips. All detected probe point values on the chip were plotted. The* central diagonal lines* were used to classify gene expression levels into three groups: group I, >twofold change increase in gene expression; group II, gene expression levels within a twofold change; and group III, >twofold change decrease in gene expression

The expression profiles of all the samples passed the microarray quality control (Table 3); a scatter plot was constructed with a two-dimensional rectangular coordinate plane (Fig. 1).

**Table 3:** Sample qualification

Sample ID	OD260/280	OD260/230	Concentration (ng/µL)	RIN	28S/18S	Results
A	2.06	1.9	1,126.89	8.9	1.8	Qualified
B	2.06	2.09	1,692.74	8.7	1.9	Qualified
C	2.06	2.19	1,092.17	8.0	1.8	Qualified
1	2.07	1.95	1,431.45	8.6	1.7	Qualified
2	2.07	2	1,428.15	8.5	1.8	Qualified
3	2.07	2.14	1,024.49	8.9	2.1	Qualified

**Fig. 2:**
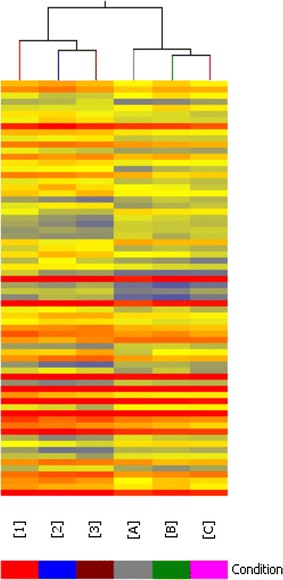
Hierarchical clustering map of DEGs. The result of hierarchical clustering on conditions shows a distinguishable gene expression profiling among samples

**Fig. 3:**
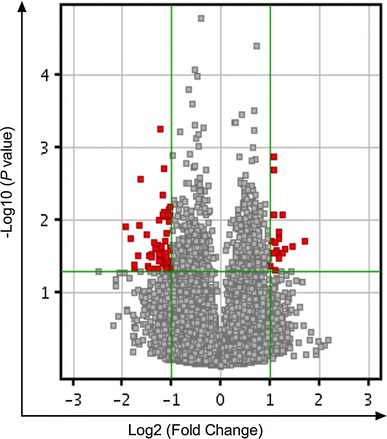
Volcano plot of DEGs. The *vertical lines* correspond to twofold up and down, respectively, and the *horizontal line* represents a *P* value of 0.05. So the *red point* in the *plot* represents the differentially expressed genes with statistical significance

Using hierarchical clustering map analysis with probe sets, the DEGs were identified in visualization, which readily distinguished the 2 groups as shown in Fig. 2. The volcano plot of DEGs revealed a total of 49 probe sets that showed a ≥2.0-fold change and *P* value ≤0.05, as shown in Fig. 3. Of 49 genes, 14 genes were found to be up-regulated and 35 genes down-regulated (Table 4).

**Table 4:** Differentially expressed genes at least twofold higher

Gene symbol	GenBank accession	Description	Fold change	*P* value
Up-regulated genes				
*GCET2*	NM_001008756	Germinal center expressed transcript 2	3.25461	0.01955
*TMEFF1*	NM_003692	Transmembrane protein with EGF-like and two follistatin-like domains 1	2.48936	0.02476
*PTTG3*	NR_002734	Pituitary tumor-transforming 3 on chromosome 8	2.39659	0.00859
*CFTR*	NM_000492	Cystic fibrosis transmembrane conductance regulator isoform 36	2.37741	0.02794
*MS4A6A*	NM_022349	Membrane-spanning 4-domains, subfamily A, member 6A	2.32408	0.02803
*FFAR2*	NM_005306	Free fatty acid receptor 2	2.26184	0.01446
*BX648831*	BX648831	Poly(A) binding protein, cytoplasmic 4-like	2.25877	0.01799
*GPRC6A*	NM_148963	G-protein-coupled receptor, family C, group 6, member A	2.17761	0.02608
*SLC25A42*	NM_178526	Solute carrier family 25, member 42	2.13807	0.02038
*SVEP1*	AK027870	Sushi, von Willebrand factor type A, EGF and pentraxin domain containing 1	2.1106	0.00132
*NLRP4*	NM_134444	NLR family, pyrin domain containing 4	2.10449	0.00205
*GLP1R*	NM_002062	Glucagon-like peptide 1 receptor	2.09506	0.02839
*LOC643406*	BC031676	Hypothetical protein LOC643406	2.04176	0.01908
*FLJ14816*	BC113708	Hypothetical protein FLJ14816	2.01485	0.04223
Down-regulated genes				
*RPL28P1*	XR_019242	Ribosomal protein L28 pseudogene 1	3.80897	0.01208
*RPL23A*	NM_000984	Ribosomal protein L23a	3.35458	0.04575
*RPL13AP3*	BC067891	Ribosomal protein L13a pseudogene 3	3.0956	0.00275
*COX19*	NM_001031617	COX19 cytochrome c oxidase assembly homolog (S. cerevisiae)	2.85475	0.01561
*RBMX*	NM_002139	RNA binding motif protein, X-linked	2.77763	0.04326
*LOC341412*	CA455253	Hypothetical LOC341412, pseudo gene	2.764	0.0313
*LOC641784*	AW302767	Similar to ribosomal protein L31, pseudo gene	2.7233	0.04737
*FOXP1*	NM_032682	Forkhead box P1	2.63004	0.03159
*COL27A1*	NM_032888	Collagen, type XXVII, alpha 1	2.62832	0.02947
*PTMA*	NM_002823	Prothymosin, alpha	2.58023	0.04672
*CALCOCO2*	NM_005831	Calcium binding and coiled-coil domain 2	2.53815	0.02005
*DNAJB6*	NM_005494	DnaJ (Hsp40) homolog, subfamily B, member 6	2.53533	0.02531
*LOC391560*	XR_018524	Ribosomal protein L32 pseudogene 7	2.43023	0.04685
*ZNF234*	NM_006630	Zinc finger protein 234	2.37866	0.0357
*WASF2*	NM_006990	WAS protein family, member 2	2.37554	0.02993
*AP3S2*	NM_005829	Adaptor-related protein complex 3, sigma 2 subunit	2.36803	0.01003
*KLF2*	NM_016270	Kruppel-like factor 2 (lung)	2.36563	0.03192
*ZC3H11A*	NM_014827	Zinc finger CCCH-type containing 11A	2.35468	0.02414
*RPS16P9*	XR_016930	Ribosomal protein S16 pseudogene 9	2.32731	0.00056
*EIF1B*	NM_005875	Eukaryotic translation initiation factor 1B	2.28022	0.00834
*NR4A2*	NM_006186	Nuclear receptor subfamily 4, group A, member 2	2.25709	0.03943
*LY6G6C*	NM_025261	Lymphocyte antigen 6 complex, locus G6C	2.19288	0.0412
*RPS7P5*	AK098605	Ribosomal protein S7 pseudogene 5	2.17098	0.00883
*RPLP0P2*	NR_002775	Ribosomal protein, large, P0 pseudogene 2	2.15877	0.03239
*CCDC144A*	BC034617	Coiled-coil domain containing 144A	2.15257	0.01531
*GARS*	NM_002047	Glycyl-tRNA synthetase	2.14073	0.04554
*LOC388524*	NM_001005472	Similar to Laminin receptor 1	2.13836	0.04665
*ZNF467*	NM_207336	Zinc finger protein 467	2.12557	0.04513
*LOC732186*	XR_016076	Similar to signal sequence receptor gamma subunit, pseudo gene	2.0591	0.01044
*ZBTB43*	NM_014007	Zinc finger and BTB domain containing 43	2.05301	0.02564
*RPL13AP23*	XR_018808	Ribosomal protein L13a pseudogene 23	2.04535	0.00653
*RPLP1P7*	CH471086	Ribosomal protein, large, P1 pseudogene 7	2.04394	0.00846
*RPL31P10*	XR_018695	Ribosomal protein L31 pseudogene 10	2.04042	0.03652
*SNX29*	AK024473	Sorting nexin 29	2.03297	0.04401
*LOC648361*	XM_001127349	Similar to 40S ribosomal protein S12, pseudogene	2.00295	0.04577

### Validation of gene expression results by using quantitative RT-PCR

**Fig. 4:**
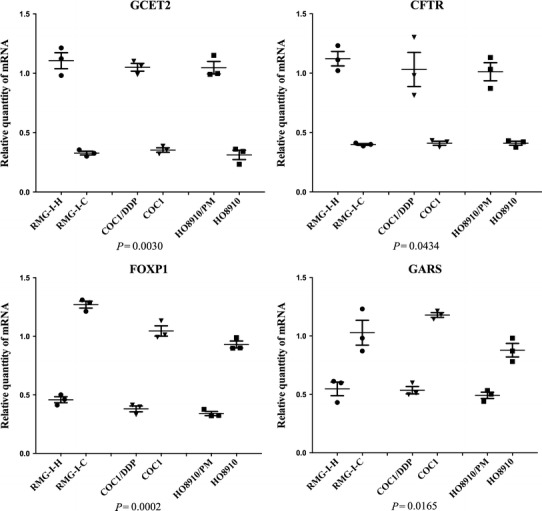
Quantitative real-time PCR validation for 4 selected genes. Quantitative real-time PCR for selected genes (*GCET2, NLRP4*, *FOXP1* and *SNX29)* found to be differentially expressed in gene microarrays. The relative expression of *GCET2* and *CFTR* was significantly higher in RMG-I-H, COC1/DDP, HO8910/PM than RMG-I-C, COC1, HO8910, respectively. The relative expression of *FOXP1* and *GARS* was significantly lower in RMG-I-H, COC1/DDP, HO8910/PM than RMG-I-C, COC1, HO8910, respectively. (*P* < 0.05, one-way ANOVA)

Four highly differentially expressed genes (i.e., *GCET2, CFTR*, *FOXP1* and *SNX29*) were selected for quantitative RT-PCR analysis as shown in Fig. 4. These results were in good agreement with the microarray data, confirming the reliability of the microarray results.

### Validation of protein expression by immunohistochemical staining

**Fig. 5:**
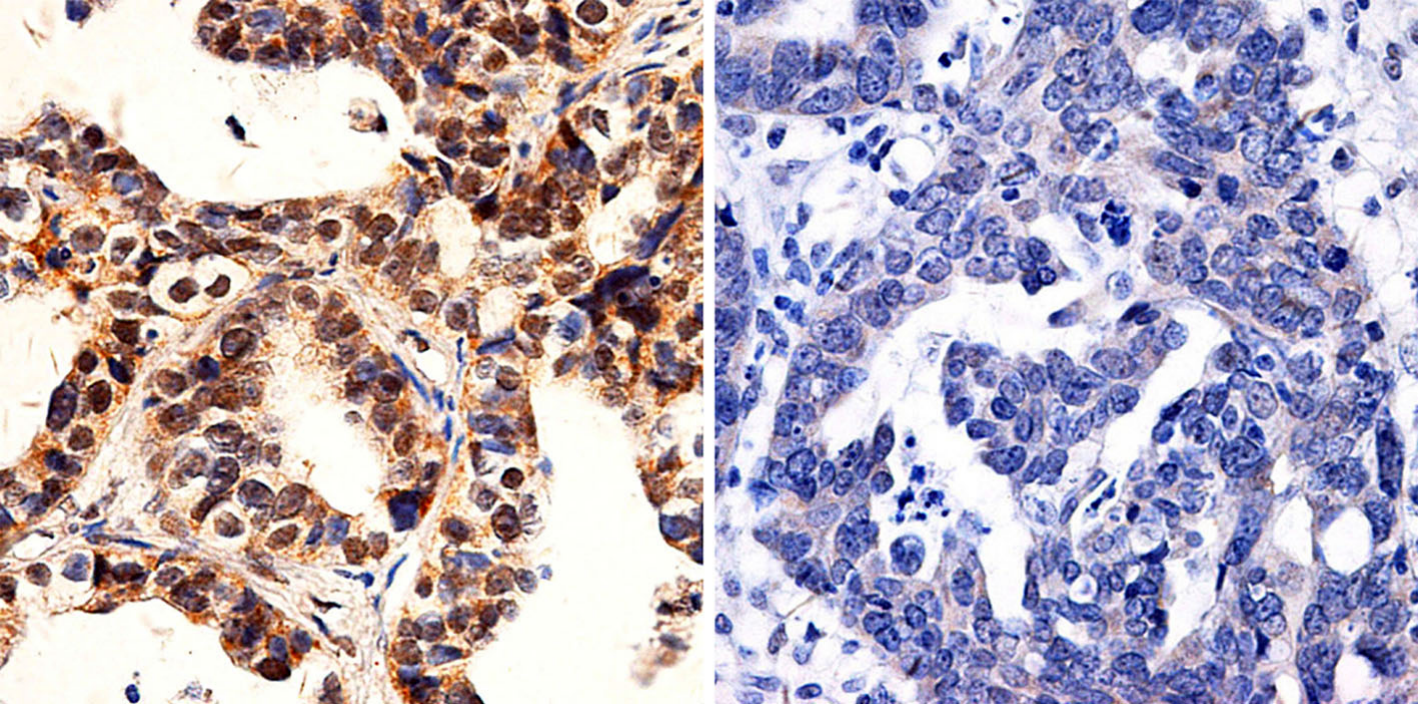
Immunohistochemical staining for FOXP1. Representative immunohistochemical staining for FOXP1. *Left panel* chemotherapeutic sensitive sample shows a positive nuclear staining for FOXP1. *Right panel* chemotherapeutic resistant sample displays a negative nuclear staining for FOXP1

**Fig. 6:**
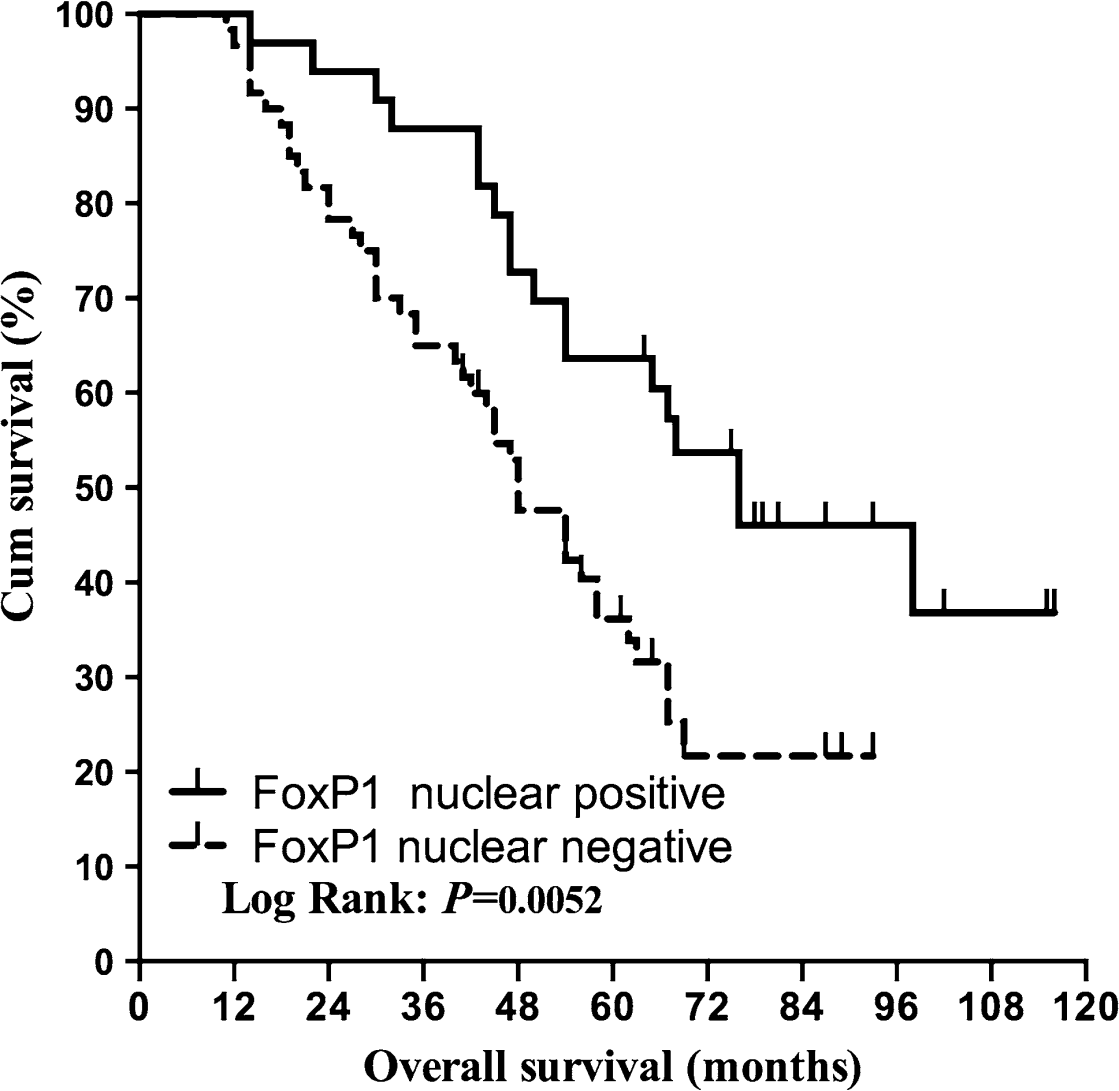
Kaplan–Meier survival analysis of chemotherapic ovarian cancer patients. Kaplan–Meier survival analysis shows that the positive nuclear staining of FOXP1 is an independent risk factor in ovarian cancer patients and strongly correlates with good prognosis

To confirm gene expression results at the protein level, immunohistochemistry for FOXP1 was carried out on all paraffin embedded samples. For metastasis, as shown in Table 5, FOXP1 nuclear positive staining in EOC was detected in 12 of 29 EOC samples (41.4 %), while only 4 of 25 OM samples (16.0 %) showed positive nuclear staining for FOXP1 (*P* = 0.042). For chemotherapeutic resistance, as shown in Table 6 and depicted in Fig. 5, FOXP1 nuclear positive staining in sensitive group was detected in 17 of 40 sensitive samples (42.5 %), while only 5 of 30 resistant samples (16.7 %) showed positive nuclear staining for FOXP1 (*P* = 0.021). A Kaplan–Meier survival analysis was applied to further investigate the effect of FOXP1 protein on ovarian cancer patients, as shown in Fig. 6, positive nuclear staining of FOXP1 was an independent risk factor and strongly correlated with prognosis.

**Table 5:** FOXP1 protein expression in 29 primary epithelial ovarian cancer (EOC) and 25 omental metastasis (OM) epithelial ovarian cancer

	Cases	Nucleus staining	
		−	+
EOC	29	17	12
OM	25	21	4

**Table 6:** FOXP1 protein expression in 40 chemotherapy-sensitive epithelial ovarian cancers and 30 resistant ovarian epithelial cancers

	Cases	Nucleus staining	
		–	+
Sensitive	40	23	17
Resistant	30	25	5

## GO function analysis and Signal pathway result of differential genes

Significant bioprocesses of the DEGs, gene expression, biopolymer biosynthetic process, macromolecule biosynthetic process, cAMP-mediated signaling, nucleic acid metabolic process, transcription and so on (Table 7). A total of 176 KEGG pathways were enriched for the 49 DEGs, including 20 significantly enriched pathways (Table 8), such as P450 hydroxylations, HIF-1-alpha transcription factor network, mechanism of acetaminophen activity and toxicity, cystic fibrosis transmembrane conductance regulator (CFTR) and beta 2 adrenergic receptor (B2AR) pathway, alpha6beta4integrin, negative regulation of the PI3K/AKT network.

**Table 7:** Classification of the up-regulated and down- regulated genes involved in the significant bioprocesses

GO Term	*P* value	Count in selection	% Count in selection	Count in total	% Count in total
Gene expression	9.36E−04	11	39.285713	2,187	14.157172
Biopolymer biosynthetic process	0.001659173	10	35.714287	1,972	12.765407
Macromolecule biosynthetic process	0.0031013	10	35.714287	2,141	13.859399
cAMP-mediated signaling	0.00382362	2	7.142857	51	0.33013982
Nucleobase, nucleoside, nucleotide and nucleic acid metabolic process	0.00942933	10	35.714287	2,495	16.150957
Cyclic-nucleotide-mediated signaling	0.010541212	2	7.142857	86	0.55670637
Transcription	0.014451807	7	25	1,483	9.599948
Biosynthetic process	0.014984685	10	35.714287	2,668	17.270844
G-protein-coupled receptor activity	0.016115764	4	14.285714	546	3.5344381
Translation	0.021661116	3	10.714286	332	2.1491456
Nucleic acid binding	0.030367257	10	35.714287	2,970	19.22579
Biopolymer metabolic process	0.030960111	12	42.857143	3,891	25.187727
Receptor activity	0.036230754	6	21.428572	1,398	9.049715
G-protein-coupled receptor protein signaling pathway	0.036383796	4	14.285714	702	4.5442777
Second-messenger-mediated signaling	0.042707212	2	7.142857	182	1.178146
Ribonucleoprotein complex	0.046174083	3	10.714286	447	2.8935785
Rhodopsin-like receptor activity	0.04668641	3	10.714286	449	2.9065251
Structural constituent of ribosome	0.0483463	2	7.142857	195	1.2622993
Macromolecule metabolic process	0.049876012	13	46.42857	4,646	30.07509

**Table 8:** Analysis of the differential gene pathways

Pathway	Number of entities	Matched with technology	Matched with entity list	*P* value
P450 Hydroxylations	19	4	2	0.002811053
HIF-1-alpha transcription factor network	88	73	6	0.002937696
Mechanism of acetaminophen activity and toxicity	12	5	2	0.004168975
Hypoxic and oxygen homeostasis regulation of HIF-1-alpha	111	86	6	0.006581206
Cystic fibrosis transmembrane conductance regulator (CFTR) and beta 2 adrenergic receptor (B2AR) pathway	14	3	1	0.006656855
De novo synthesis of IMP	32	4	1	0.011070482
Cytochrome p450	54	9	2	0.01444615
Phase 1 functionalization	87	9	2	0.01444615
il12 and stat4 dependent signaling pathway in th1 development	13	10	2	0.015267268
alpha6beta4integrin	53	50	4	0.01597487
IL4-mediated signaling events	84	52	4	0.018223463
Purine metabolism	100	9	1	0.022020191
Xenobiotics	60	15	2	0.0333969
Stathmin and breast cancer resistance to antimicrotubule agents	18	2	1	0.03385132
Negative regulation of the PI3 K/AKT network	12	2	1	0.03385132
TCR	140	125	6	0.035289083
Gap-filling DNA repair synthesis and ligation in GG-NER	7	2	1	0.038461793
Gap-filling DNA repair synthesis and ligation in TC-NER	7	2	1	0.038461793
FOXA1 transcription factor network	53	40	3	0.04229567
Nucleotide metabolism	198	22	1	0.049933493

## Establishment of regulatory network for the DEGs

**Fig. 7:**
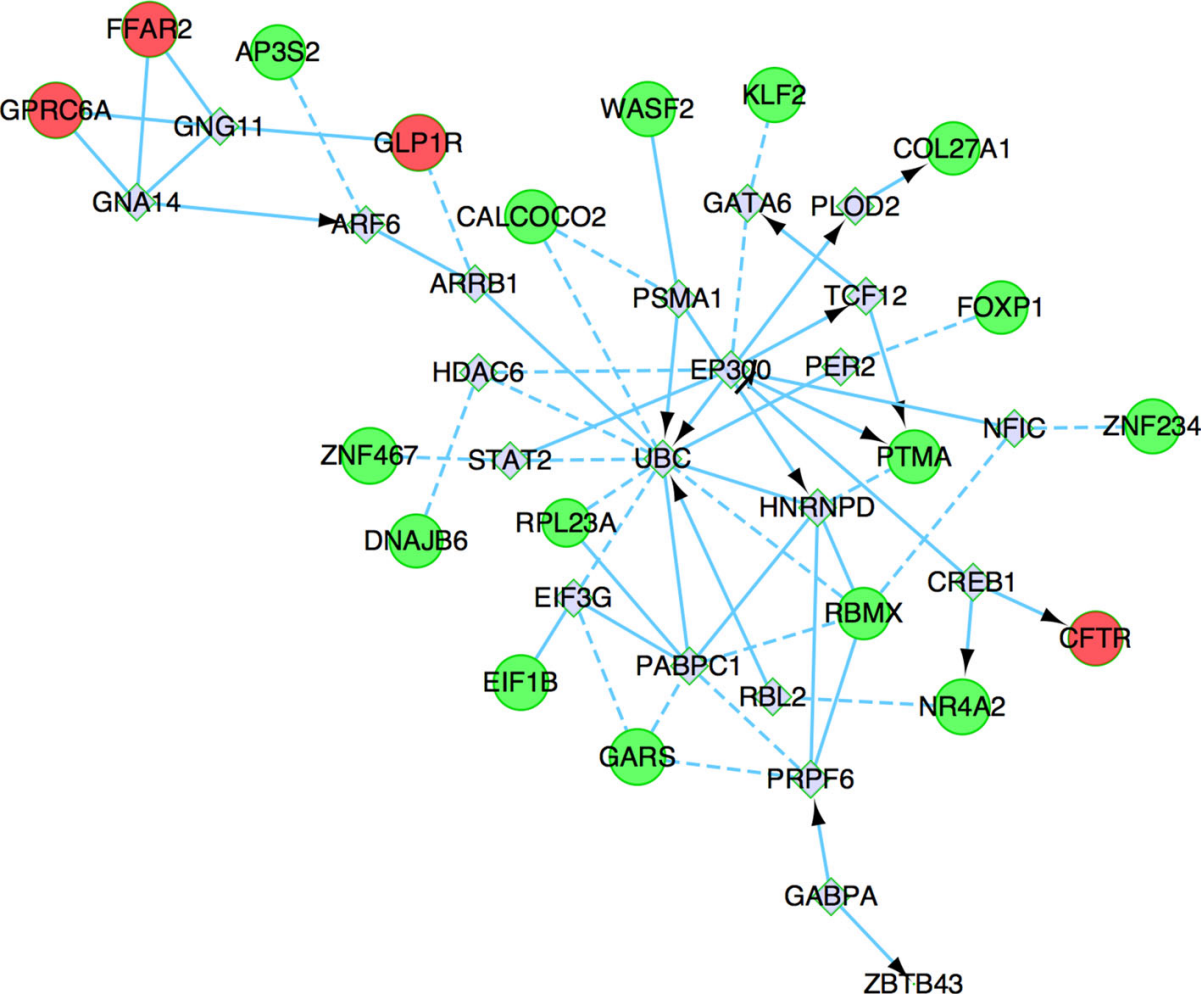
Interaction network of the differentially expressed gene. Genes with more links are shown in bigger size. Proteins shown in *red* are encoded by up-regulated genes, while those in *green* are encoded by down-regulated genes, the *gray* represents the predicted genes. *Arrow line* represents definite control relationship, *dotted line* represents predicted control relationship, *solid line* represents inhibition

In order to further investigate the global expression occurring and to define how individual up- or down-regulated genes interact with each other to have a coordinated role, we identified potential networks for these DEGs (Fig. 7). Among the 49 DEGs, 20 were involved in the establishment of regulation network, of which 4 were up-regulated and 16 were down-regulated. A total of 21 transcription factors (TFs) were predicted, in which *UBC* and *EP200* are the most connected predicted hub genes.

## Discussion

Ovarian cancer has the highest mortality rate of all gynecologic cancers, and 75 % of patients diagnosed with ovarian cancer are already at an advanced stage. Chemotherapy is important in treating and preventing the recurrence of ovarian cancer; however, resistance is an obstacle to overcome and finding new treatment strategies has become increasingly valuable. High-throughput technologies for assaying gene expression, such as high-density oligonucleotide and cDNA microarrays, may offer the potential to identify clinically relevant genes highly differentially expressed between different cell lines. Thus, this study showed the first communication of an investigation that involved the genome-wide examination of differences in gene expression between ovarian cancer cell lines and their sub-lines with enhanced metastasis and chemotherapic resistance. We identified 49 genes that were expressed differentially between Group A, B, C and Group 1, 2, 3, and the average change in expression level between the two groups was at least twofold. The known functions of some of these genes can provide insights with the highly metastasis and chemotherapic resistance for ovarian cancer, although others are still useful for a further research.

*GCET2* is found to be the most up-regulated genes in the more enhanced metastasis and chemoresistance cell group, and it is also known as human germinal center associated lymphoma (*HGAL*) gene, is specifically expressed in germinal center B-lymphocytes and germinal center-derived B cell lymphomas [12], but its function is largely unknown. The *GCET2* gene is located on chromosome 3q13 and encodes a 178-amino acid (aa) protein with 51 % identity and 62 % similarity to the murine M17 protein [13]. GCET2 is a cytoplasmatic protein that may also associate with cell membrane. *GCET*2 expression is associated with improved survival in diffuse large B cell lymphoma (DLBCL) and classic Hodgkin lymphoma patients [14]. In vitro studies in human lymphocytes demonstrated that *HGAL* increased the binding of myosin to F-actin and inhibits the ability of myosin to translocate actin by reducing the maximal velocity of myosin head/actin movement [15]. In vitro *HGAL* enhances BCR signaling by binding and increasing Syk activation, in vivo older *HGAL* transgenic animals progressively developed polyclonal lymphoid hyperplasia and reactive AA amyloidosis [16], these finding suggests that *GCET2* may play a role in humoral immune responses. No articles about expression and function of *GCET2* on ovarian tissue have been published until now, and our findings make a possible insight of this gene in the study of ovarian cancer, especially about the aspects of metastasis and chemoresistance.

Cystic fibrosis transmembrane conductance regulator (*CFTR*, *ABC35* or *ABCC7*) was found among the most up-regulated genes in more metastasis and chemoresistance cell line group, and it participates in the beta-adrenergic-dependent CFTR expression pathway. Loss of function mutations of this gene causes the autosomal recessive lethal disease cystic fibrosis (CF) and congenital bilateral aplasia of the vas deferens. There is an increasing interest in the association of cancer incidence with the genetic variations in the *CFTR* gene. Large cohort studies in North American and European patients with CF found that there was a marked increase in the risk of malignancies affecting the gastrointestinal tract, even to 17 times higher risk of digestive cancer with most cases arising in the bowel [17]. Meanwhile, mutations and low-penetrance polymorphisms in the *CFTR* gene have been found in patients with various cancers, including pancreatic cancer [18], breast cancer [19], cervical cancer [20], melanoma [21], prostate cancer [22] and lung cancer [23, 24]. On the other hand, *CFTR* has been suggested to interact with various cancer-related kinases [25]. It encodes a member of the ATP-binding cassette (*ABC*) transporter superfamily. *ABC* proteins transport various molecules across extracellular and intracellular membranes. *ABC* genes are divided into seven distinct subfamilies (*ABC1*, *MDR/TAP*, *MRP*, *ALD*, *OABP*, *GCN20*, *White*). Meanwhile, *CFTR* is a member of the *MRP* subfamily that is involved in multidrug resistance. The encoded protein is a cAMP-activated Cl^−^ channel lining the luminal/apical surfaces of epithelial cells in airway, gut, and exocrine glands, and there is a functional coupling between *CFTR* and *MRP2* that may be mediated by PDZ protein [24]. Taken together, our gene expression profile that show a significant up-regulated result of *CFTR* in more metastasis and chemoresistant ovarian cancer cell lines are consistent with previous findings, a further research on the mechanism of *CFTR* on ovarian cancer or selective inhibition of *CFTR* or its pathway may give a insight in therapeutic effects against metastatic and chemoresistant of ovarian cancer.

*RBMX* gene, also known as *HnRNP G*, is a member of heterogeneous nuclear ribonucleoprotein (hnRNP) family and can collaborate with *hTra2*-*beta1* (human transformer-2-beta1) as sequence-specific transacting factors to exert antagonistic effects on alternative splicing which is recognized as a pivotal mechanism in regulation of gene expression and associated to tumorigenesis and metastasis of a wide variety of human cancers [26]. It is proposed that the ratio of hnRNP G/hTra2-beta1 influenced cellular splicing preference [27, 28]. Some researches revealed that hnRNP G-protein showed as tumor suppressor in endometrial carcinoma [27] and oral squamous cancer [29], its activity was elicited by transactivating tumor suppressor Txnip gene [30]. A recent research showed that high frequency of hnRNP G-protein reduction and loss of expression in precancerous and human oral squamous cell carcinoma tissue specimens, suggesting that reduction in hnRNP G may play an important role in the early pathogenesis of oral squamous cell carcinomas [31].

*GPRC6A* encodes an orphan G-protein-coupled receptor, mediates the non-genomic effects of testosterone and other anabolic steroids in multiple tissue, and it is a potential target for developing antagonists and agonists that would have broad applications in health and disease [32], including cancer. A genome-wide association study on prostate cancer identified *GPRC6A* was one of the five novel genetic loci associated with prostate cancer in Japanese and Chinese Han population [33, 34], and the same result was verified by a genome-wide testing of putative functional exonic variants in a multiethnic population [35]. *GPRC6A* is expressed at higher levels in human prostate cancer cells and prostate cancer tissues and small interfering RNA knockdown of *GPRC6A* attenuates these response in human prostate cancer cell lines [36]. *GPRC6A* is also coupled to signaling pathways, such as phosphatidylinositol 3-kinase that are known to be deregulated in prostate cancer [32]. On all accounts, nearly all researches about *GPRC6A* associated with cancer focused on prostate cancer, no investigation about this gene on ovarian cancer has been published.

*FOXP1*, as one of the down-regulated genes, drew our attention for a further research. *FOXP1* is a *FOX* family member consisting of the winged-helix DNA-binding domain and the N-terminal transcriptional repression domain, and it is widely expressed and plays a key role in the development of various human tissues [37, 38]. *FOXP1* represses its target genes by forming homodimers or heterodimers with *FOXP2* and *FOXP4* [39], it has been suggested to be both a tumor suppressor candidate and potential oncogene, because of its differential expression levels in distinctive types of tumors, including B cell lymphomas [40], breast cancer [41, 42], endometrial cancer [43], prostate cancer [44], non-small cell lung cancer [38] and renal cell carcinoma [45], the loss of *FOXP1* in breast cancer has been associated with shorter survival times [42]. Until now, no article about *FOXP1* expression in ovarian cancer has been published, and we made the first investigation of *FOXP1* protein expression in ovarian tissue and found that nuclear staining of *FOXP1* decreased as the metastasis increased, a significant decrease in *FOXP1* expression in the resistance group, nuclear *FOXP1* expression were independent risk factors strongly correlated with prognosis of ovarian cancer, above all, *FOXP1* may serve as a good marker for late stage ovarian cancer and chemoresistance EOC patients, high expression of *FOXP1* in nucleus is associated with improved survival in patients with ovarian cancer.

There are some pseudogenes which shows significant expression difference in enhanced metastasis and chemoresistant ovarian cancer cell lines, in which *BC031676* and *BC113708* are up-regulated, and *RPL28P1*, *RPL23A*, *RPL13AP3*, *LOC341412*, *LOC641784*, *LOC391560*, *RPS16P9*, *LOC732186*, *RPL13AP23*, *RPLP1P7*, *RPL31P10*, *LOC648361* are down-regulated, and most of them are ribosomal protein pseudogenes. Pseudogenes are DNA sequences similar to genes encoding functional proteins but are presumed to be nonfunctional due to mutations and truncation by premature stop codons [46]. Ribosomal protein (RP) pseudogenes constitute the largest family of pseudogenes (approximately 2000 RP processed pseudogenes), and they are constitutively expressed at reasonably stable levels and are very highly conserved [47]. Although pseudogenes have long been considered as nonfunctional genomic sequences, during recent two decades, especially with the broad applications of next-generation sequencing technologies, emerging evidences have confirmed that some pseudogenes have acquired diverse functions in regulating development and diseases, especially in cancers [48]. Some pseudogenes are specifically expressed in certain cancers or diseases. It has been shown that the pseudogene of *PTEN*, *PTENP1*, was selectively lost in some human cancer cells, resulting in decreased expression of *PTEN* and abnormal proliferation of cancer cells [49]. The expression of *MYLKP1*, a duplicated pseudogene of *MYLK*, can decrease the stability of *MYLK* mRNA at the posttranscriptional level and stimulate cell proliferation [50]. Recently, a study provided a systematic approach to analyze expressed pseudogenes, enabling comparisons of cancer versus benign tissues in multiple solid tumors, which overcome the limitations of previous analyses of pseudogene expression. They observed 218 pseudogenes expressed only in cancer samples, of which 178 were observed in multiple cancers, and 40 were found to have highly specific expression in a single cancer type only, finally they described *ATP8A2*-*J* and *CXADR*-*J* pseudogenes preferentially associated with distinct subsets of breast cancer and prostate cancer patients, respectively [51]. Besides cancers, pseudogenes also involve in the development of other diseases, such as *HMGA1*-*p* [52]. Although the regulatory functions of pseudogenes seem to be striking, the functional studies of pseudogenes are still in its early stage. The pseudogenes in our study should not be useless, their functions and relationships with ovarian cancer, especially the enhanced metastasis and chemoresistance should be investigated in near further.

In conclusion, this study has identified potential DEGs responsible for enhanced metastasis and chemoresistance in ovarian cancer cell lines. Among the 49 DEGs, 14 genes were up-regulated and 35 genes were down-regulated. Prospective investigations using a combination of genomic and proteomic approaches are required to validate the functionality of these targets identified.
